# Substrate Concentration Modulates Peptide Profile and Bioactivity in Alcalase Hydrolysis of Bovine Plasma Proteins at Fixed Enzyme-to-Substrate Ratio

**DOI:** 10.3390/molecules31152693

**Published:** 2026-08-03

**Authors:** Omar A. Figueroa-Moreno, José E. Zapata-Montoya, Ismael Marcet, Manuel Rendueles, Natalia Díaz Fernandez

**Affiliations:** 1 Faculty of Engineering, University of La Guajira, Riohacha 440002, Colombia; 2 Department of Food, Faculty of Pharmaceutical and Food Sciences, University of Antioquia, Carrera 51, Number 62-13, Medellin 050010, Colombia; edgar.zapata@udea.edu.co; 3 Department of Chemical and Environmental Engineering, University of Oviedo, C/Julián Clavería 8, 33006 Oviedo, Spain; marcetismael@uniovi.es (I.M.); mrenduel@uniovi.es (M.R.); 4 Department of Physical and Analytical Chemistry, University of Oviedo, C/Julián Clavería 8, 33006 Oviedo, Spain; diazfnatalia@uniovi.es

**Keywords:** bovine plasma, Alcalase, hydrolysis, substrate concentration, antioxidant, antihypertensive

## Abstract

Many studies have been conducted testing different enzymes and protein combinations to produce peptides with different types and levels of bioactivities; however, their effect on peptide sequence, peptide size distribution, and bioactivity, while maintaining a constant enzyme–substrate ratio, has been less frequently addressed. In this research, bovine plasma has been hydrolysed by Alcalase at several substrate concentrations (5, 10, 15, and 20 g/L) and at a fixed enzyme–substrate ratio of 1:25 (*w*/*w*, Alcalase mass per plasma protein mass). For each substrate concentration, the in vitro antioxidant and antihypertensive properties of the hydrolysates produced were assessed. In addition, the molecular weight distribution of the peptides and their sequence were also analysed by SE-HPLC and RP-UPLC-MS/MS, respectively. According to the results obtained, with the lowest substrate loading producing the highest proportion of small peptides: at DH 19%, peptides below 3 kDa represented 45% of the chromatographic area at 5 g/L and 35% at 20 g/L. The hydrolysate obtained at 20 g/L showed the highest antioxidant activity, with ABTS and ORAC values of 1833.98 and 1461.36 μmol TE/g protein, respectively, compared with 1493.13 and 1071.63 μmol TE/g protein for the hydrolysate obtained at 5 g/L. In contrast, the 5 g/L hydrolysate showed the highest ACE inhibitory activity (53.73 ± 3.08%) and the lowest IC50 value (0.41 mg/mL), whereas the 20 g/L hydrolysate showed 35.96 ± 1.55% inhibition and an IC50 of 0.86 mg/mL. These results indicate a trade-off between antioxidant and antihypertensive performance and demonstrate that substrate loading, evaluated under a fixed enzyme-to-substrate ratio, is an effective variable for modulating peptide size distribution, the peptide profile and the resulting bioactivity of bovine plasma hydrolysates.

## 1. Introduction

Bovine blood is a byproduct of the meat industry, produced in large quantities and of low industrial value but high in protein. In particular, the plasma fraction can be easily separated from the red fraction by centrifugation and accounts for about 60–70% of the total blood volume, containing approximately 8% of the total bovine blood protein. The main proteins in bovine plasma include albumins, immunoglobulins, α- and β-globulins, and fibrinogen, all of which have a significant bioactive potential and whose hydrolysates are an important source of antioxidant and antihypertensive peptides [1,2]. In this regard, several authors have found novel bioactive peptides by hydrolysing plasma proteins from livestock blood using a variety of enzymes, such as papain [3], pepsin and trypsin [4], and Alcalase^®^ [5]. In most of these studies, authors have found novel bioactive peptides using new enzymes capable of releasing new peptide sequences from plasma proteins or previously known enzymes that had never been used to produce and characterize bioactive peptides from this protein source. In this respect, it is important to highlight the use of Alcalase for this purpose. Alcalase, a product commercialized by Novozymes Corp., is formulated with 50% (*w*/*w*) glycerol, 41% (*w*/*w*) water, and 9% (*w*/*w*) protease extract from Bacillus licheniformis. This protease extract is able to recognize a wide variety of amino acids positioned at P1 and P2′ or P3′, so it shows broad specificity and enzyme selectivity, being able to hydrolyse a large variety of protein substrates and yielding a hydrolysate with a high content of small peptides [6]. Considering that the smallest amino acid sequences, with molecular weights between 1 and 4 kDa, are the most interesting for nutritional and/or pharmaceutical uses [7], especially as functional ingredients due to the relevant antioxidant capacity reported for peptides < 3 kDa [8] and their advantages in intestinal absorption [9], their production is of particular interest. Due to its high hydrolytic efficiency, the use of Alcalase to produce bioactive peptides has proven to be very effective.

Alternatively, the production of new bioactive peptides from proteins can be studied by considering not only the enzyme used or the protein source but also the effect of varying the hydrolysis conditions. Recently, some authors have reported that, by adjusting pH, temperature, and reaction time during Alcalase-mediated hydrolysis of walnut protein, peptides smaller than 3 kDa were obtained that retained strong ACE-inhibitory and antioxidant activities even after simulated gastrointestinal digestion [10,11]. Previous studies have explored the production of antioxidant and antidiabetic peptides from casein by using Protamex™, a commercial product with Bacillus proteinase activity, and varying the pH, time of hydrolysis, and temperature [12]. Moreover, Tu et al. [13] tested the effect of several enzyme/substrate ratios and hydrolysis times on the bioactivity, assessed in silico, of the peptides obtained after hydrolysing casein with trypsin. Furthermore, Ding et al. [14] optimized the production of antioxidant peptides from pea protein using Alcalase while varying the enzyme to substrate ratio, temperature, and pH.

In this regard, the effect of varying the initial substrate concentration while maintaining a constant enzyme–substrate ratio has been less frequently considered in studies aimed at producing antioxidant and antihypertensive peptides, which more often evaluate enzyme dosage and other hydrolysis conditions such as pH and temperature. The initial substrate concentration is not only a reaction variable but also a process-design variable [15]. Higher substrate loadings are attractive because they increase volumetric productivity and can facilitate downstream operations such as spray drying by reducing the amount of water that must be removed [16] whereas lower loadings may improve processability in membrane-based separation systems by reducing viscosity and fouling-related limitations [17].

Although studies performed at a constant enzyme concentration and variable E:S ratio are also valid and are frequently used when enzyme dosage is treated as an economic or optimization variable, that approach simultaneously changes substrate loading and the E:S ratio. In the present work, the E:S ratio was kept constant to evaluate the effect of substrate concentration under the same enzyme dosage criterion per unit of protein. This is important because, in protein hydrolysis systems, substrate concentration may affect not only the overall hydrolysis rate but also the apparent selectivity of cleavage, the peptide profile, and bioactivity. Under this design, differences among treatments cannot be attributed to deliberate changes in the relative enzyme supply, while allowing assessment of how the absolute concentration of the hydrolysis system influences peptide generation, peptide profile, and bioactivity.

In relation to the hydrolysis of bovine plasma with Alcalase, if the goal is to produce peptide fractions of specific interest using this cheap protein source and this enzyme, it is essential to understand how protein hydrolysis phenomena affect the production of these peptides and how this effect is influenced, not only by the already widely studied operating conditions, such as pH and reaction temperature, but also by the levels of substrate loaded in the reactor [18].

Accordingly, substrate concentration deserves specific evaluation not only from a mechanistic perspective but also from a technological one. Therefore, this study aimed to analyze the influence of the initial protein concentration, while maintaining a fixed E:S ratio as a proportional enzyme-dosing criterion, on peptide generation, the peptide profile, and the antioxidant and antihypertensive properties of the hydrolysates obtained from bovine plasma hydrolysis with Alcalase. For that purpose, the degree of hydrolysis, the size of the peptides, the antioxidant properties, and the sequence of the peptides obtained for each substrate concentration tested were studied. Finally, the potential antihypertensive properties of the peptides obtained were analysed in vitro and in silico.

## 2. Results

### 2.1. Effect of Substrate Loading at Fixed E:S Ratio on Hydrolysis

**Figure 1:**
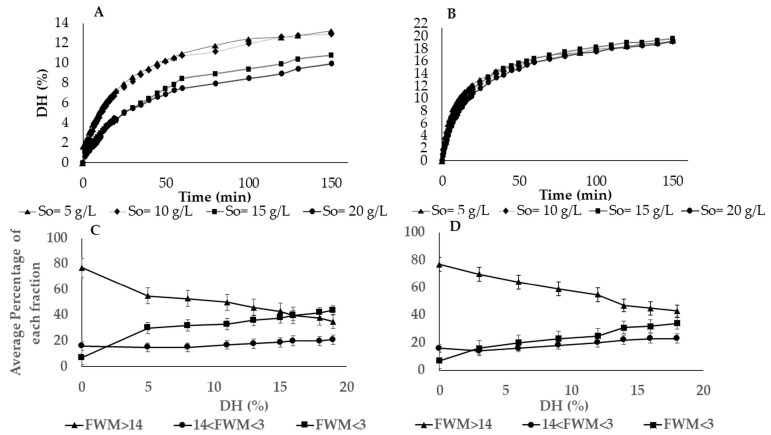
Hydrolysis curve (DH vs. time) of whole bovine plasma without dialysis (**A**) and dialyzed bovine plasma (**B**). Using Alcalase 2.9 L with different concentrations of protein (S_0_ = 5, 10, 15, 20 g/L) and an enzyme–substrate ratio of 1:25. Molecular weight distribution of enzymatic hydrolysates from bovine plasma dialyzed with Alcalase 2.9 L. Hydrolysates obtained with S_0_ = 5 g/L (**C**) and 20 g/L (**D**) and an enzyme–substrate ratio of 1:25 (*w*/*w*). Peptide size distribution is expressed as the relative area percentage of the apparent molecular weight fractions. Values were obtained from independent determinations. Error bars are shown in the plots. The average standard deviation for each fraction is indicated here. For panels (**A**) and (**B**), final DH values from independent experiments are reported in the text as mean ± SD (n = 3). For panel (**C**) (S_0_ = 5 g/L): large fraction, SDmean = ±6.2; medium fraction, SDmean = ±3.2; small fraction, SDmean = ±4.5. For panel (**D**) (S_0_ = 20 g/L): large fraction, SDmean = ±4.7; medium fraction, SDmean = ±3.0; small fraction, SDmean = ±4.9. Statistical comparisons among treatments are reported in the text.

The hydrolysis of bovine plasma with Alcalase was carried out using an E:S ratio of 1:25 (*w*/*w*, Alcalase mass per plasma protein mass) and with different initial protein concentrations. The hydrolysis curves (DH vs. time) obtained are shown in Figure 1A. In this case, the DH_max_ value decreased as the substrate concentration also decreased (Figure 1A). The final DH values obtained for whole plasma at 5, 10, 15, and 20 g/L were 13.3 ± 0.6, 12.9 ± 1.5, 10.8 ± 1.0, and 10.0 ± 1.4, respectively. In particular, significant mean differences (*p* < 0.05) were found between the DH_max_ values reached with the highest substrate concentration levels tested, 15 and 20 g/L, and the lowest substrate concentrations evaluated in this study, 5 and 10 g/L. However, there was no difference between the DH_max_ values reached with 5 and 10 g/L, nor between 15 and 20 g/L. Therefore, a change of 10 concentration units had a significant effect on the DH parameter over the time of reaction. It should be noted that no evidence of gel formation or any type of protein precipitation before or after the hydrolysis reaction was found.

The plasma was dialyzed and subsequently hydrolysed under the same conditions (Figure 1B). When the bovine plasma was dialyzed, in contrast to the previous results, no significant differences (*p* < 0.05) were found between the DH_max_ values reached for each protein concentration tested at the fixed E:S ratio selected. The corresponding final DH values for dialyzed plasma at 5, 10, 15, and 20 g/L were 19.4 ± 0.1, 19.2 ± 0.1, 19.7 ± 0.6, and 19.3 ± 0.1, respectively.

The molecular weight distribution of the peptides in the hydrolysates was analysed by dividing the chromatograms into three characteristic fractions based on their elution times. These fractions were called: the large size fraction, with peptides with a size larger than 14.0 kDa and composed mainly of intact proteins; the medium size fraction, with peptides with a size between 14.0 and 3.0 kDa; and the small size fraction, with peptides smaller than 3.0 kDa, which are those with the highest bioactive potential. The size distribution of the hydrolysates was represented by showing the percentage area for each fraction, calculated relative to the total analysis area, using the software of the HPLC device (Agilent Chemstation software, version B.03.02). According to the results obtained, at the lowest protein concentration tested, a higher rate of cleavage of the large size fraction was observed, which was compensated by a proportional increase in the medium size fraction population. In fact, at 5 g/L and at a DH value of 5%, the largest protein fraction decreased by 30% (Figure 1C), but the same fraction only decreased by 15% when the protein concentration tested was 20 g/L (Figure 1D).

### Peptide Identification in Hydrolysates Obtained at Fixed E:S Ratio

**Figure 2:**
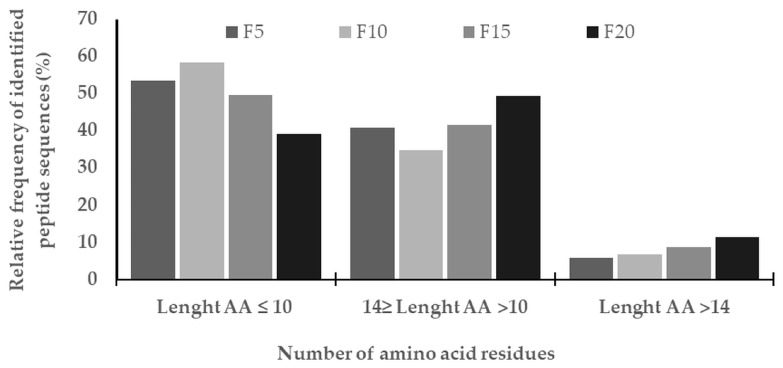
Descriptive distribution of the peptides identified in the ultrafiltered permeates (MWCO 3.0 kDa), classified according to peptide length as defined by the number of amino acid residues. F5, F10, F15, and F20 denote the permeates obtained from hydrolysates prepared at initial protein concentrations of 5, 10, 15, and 20 g/L, respectively.

To test whether peptide profiles differed when bovine plasma was hydrolysed at a fixed E:S ratio but under different initial protein concentrations, the protein concentration was adjusted to 5, 10, 15, and 20 g/L and hydrolysed with Alcalase using an E:S ratio of 1:25 (*w*/*w*, Alcalase mass per plasma protein mass) until reaching a DH value of 19% for each substrate concentration tested. The hydrolysates obtained were ultrafiltered using centrifuge tubes (MWCO of 3.0 kDa), and the peptides in the permeates were identified by RP-UPLC-MS/MS. The peptides identified in the <3 kDa permeates were grouped according to their number of amino acid residues into three classes: fewer than 10 residues, between 10 and 14 residues, and more than 14 residues. Figure 2 shows the descriptive distribution of the identified peptides among these length classes for the different hydrolysates. This descriptive analysis suggested differences in the distribution of identified peptide lengths among hydrolysates, with the hydrolysates obtained at initial protein concentrations of 15 and 20 g/L showing a lower representation of short peptides and a greater representation of longer identified sequences compared to the hydrolysates obtained with less substrate.

### 2.2. Antioxidant Activity of Bovine Plasma Hydrolysates and Relative Composition of the Amino Acids

The antioxidant properties of F5, F10, F15, and F20 were tested in vitro, and the results are shown in Table 1. All the experiments were performed before determining the protein content in the samples; therefore, the results are expressed as Trolox equivalent per g of protein. Regarding the antioxidant values measured by the ABTS method, a 20% increase in the antioxidant activity was noted when the highest and lowest substrate loads were compared (F5 and F20). Regarding the capacity to reduce the ferric iron (Fe^3+^) to ferrous iron (Fe^2+^) measured by the FRAP assay, no difference for this antioxidant capacity was detected for the assessed samples, and it would appear to be independent of the level of substrate loaded in the reactor. In addition, great differences in the moles of Trolox equivalent were recorded between the FRAP and ABTS assays for all samples, probably because food sources have a lower tendency to reduce Fe^3+^ ion than to capture ABTS and peroxyl radicals. On the other hand, the tested samples showed an increase in their oxygen radical absorbance capacity as the peptide fractions were produced with a higher initial substrate load. In fact, a 36% increase in this antioxidant ability was observed for F20 compared to F5. To obtain an integrated view of antioxidant performance, the Relative Antioxidant Capacity Index (RACI) was also calculated from the ABTS, FRAP, and ORAC data. The RACI values ranked the fractions as F15 > F20 > F10 > F5, indicating that the fractions obtained at higher substrate loadings showed the most favorable overall antioxidant profile.

**Figure 3:**
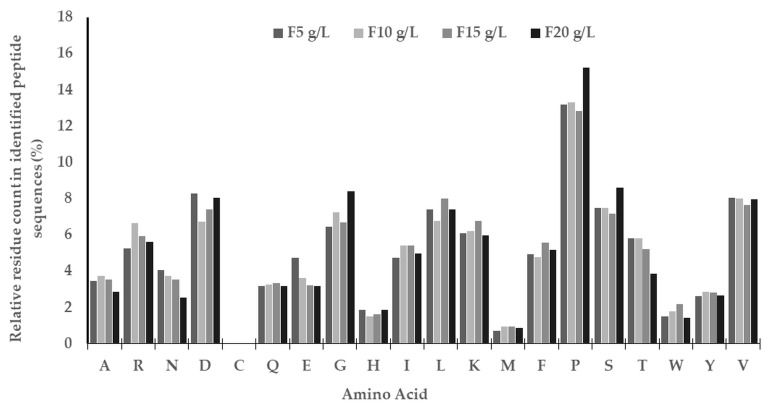
Descriptive frequency distribution of amino acid residue occurrences within the peptide sequences identified in the permeates (MWCO 3.0 kDa) obtained from bovine plasma–Alcalase hydrolysates (DH = 19%) prepared at initial protein concentrations of 5, 10, 15, and 20 g/L (F5, F10, F15, and F20, respectively). Values represent the relative frequency (%) of amino acid residues calculated from residue counts across all identified peptide sequences in each permeate, relative to the total number of residues counted in that sample.

The estimated relative distribution of amino acid residues within the peptide sequences identified in each fraction is shown in Figure 3. A broadly similar residue distribution was observed among the four hydrolysates, although F20 presented a higher relative occurrence of Pro, Gly, and Ser than the other fractions.

### 2.3. Analysis of Antihypertensive Activity of Bovine Blood Peptides

The antihypertensive activity of fractions F5, F10, F15, and F20 was evaluated in vitro, and the results are shown in Table 2. This table also includes the number of peptides identified in each fraction with AHTpin scores equal to or higher than 0.9 and below 0.9, as predicted by the AHTpin platform, a web server developed for the prediction, screening, and design of antihypertensive peptides [19]. F10 and F5 exhibited higher antihypertensive activity than F15 and F20, with no statistically significant difference between F10 and F5 or between F15 and F20 (*p* < 0.05). Accordingly, F10 presented the highest number of peptides with an SVM value equal to or higher than 0.9, followed by F5, F15, and F20.

The peptide sequences with the highest predicted antihypertensive potential (those with an SVM score of 1.7–2.8) identified in each fraction had been previously reported in [20]. Among them, F5 contained five peptides, including three with SVM scores higher than 2.0 that were not identified in the other fractions, whereas F20 contained four peptides with high predicted potential, mostly with SVM scores below 2.0.

**Figure 4:**
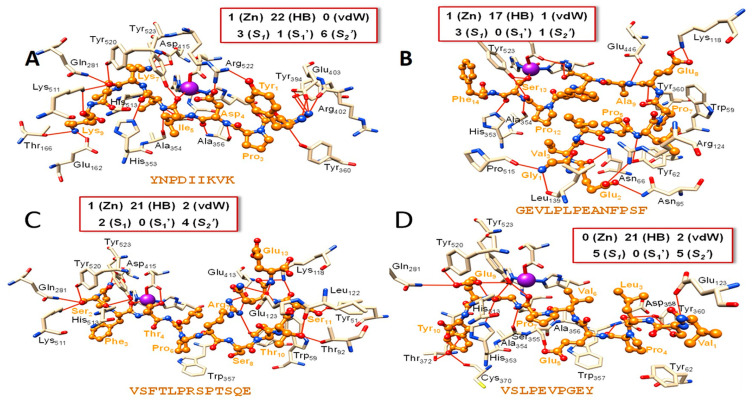
Close-up view of enzyme–ligand interactions obtained by molecular docking in ACE complexes with selected peptides (**A**): YNPDIIKVK, (**B**): GEVLPLPEANFPSF, (**C**): VSFTLPRSPTSQE, (**D**): VSLPEVPGEY). The ligand atoms are shown in ball-and-stick (C in orange, O in red, and N in blue). The number of ACE-peptide contacts is indicated for each complex (Zn: Zn-O interactions, HB: hydrogen bond, and VdW: van der Waals). The number of contacts with the ACE residues at the S1 (Ala354, Glu384, and Tyr523), S1′ (Glu162), and S2′ (Gln281, His353, Lys511, His513, and Tyr520) binding sites are also included (residue numbering as in the 1O86 PDB structure) [21]. The enzyme is shown as sticks (C in navajowhite, N in blue, O in red, and S in yellow), with the active site zinc ion in spacefilled (Zn: magenta). The ligand atoms are shown in ball-and-stick (C in orange, O in red, and N in blue).

The peptide sequences VSLPEVPGEY, GEVLPLPEANFPSF, VSFTLPRSPTSQE, and YNPDIIKVK, previously identified and reported in [20], which showed an SVM score higher than 2.0, attributed by the AHTpin in silico platform, were selected to perform the molecular coupling analysis with ACE. The molecular docking model of these four peptides in the configuration with the lowest binding energy with the ACE active site is shown in Figure 4.

## 3. Discussion

### 3.1. Effect of Substrate Loading at Fixed E:S Ratio on Bovine Plasma Hydrolysis

The biological activity of the enzymatic protein hydrolysates is at least in part related to the molecular weight of the peptides; the smallest peptides being those with the highest bioactivity [7]. In this regard, the degree of hydrolysis (DH) is a useful parameter for indirectly evaluating the molecular weight distribution of the peptides contained in the hydrolysate. Longer hydrolysis times usually result in higher DH values and in the production of smaller peptides. In this sense, whenever high DH values are required, it must be remembered that the enzymatic hydrolysis of proteins is strongly dependent on several operational parameters, among which the effect of the initial substrate concentration is a limiting parameter. In the experimental approach proposed here, substrate concentration was analyzed under a fixed E:S ratio, so that the differences observed between treatments can be discussed in terms of how protein loading modulated the hydrolysis system under the same enzyme dosage criterion, rather than under deliberate changes in enzyme dosage per unit of protein. Under this framework, changes in DH behavior are better interpreted as the combined consequence of substrate-dependent effects on the reaction environment and on the apparent progression of proteolysis.

There are reasons to affirm that changes in the concentration of the initial substrate in the hydrolysis can generate changes in the composition of the hydrolysates over the time of reaction [21]. These changes, which are due to the selectivity of the enzyme, suggest the appearance of peptides with different chemical characteristics. These peptides may inhibit the protease and influence both the rate of hydrolysis and the reaction mechanisms [20,22] and therefore the DH_max_ value obtained. However, the speed of enzymatic reactions can also be influenced by the ionic strength of the medium, which interferes with protein–protein interaction mechanisms and their thermal stability [23]. In addition, there is evidence of linear increases in the conductivity of protein solutions with substrate concentrations below 10% (*w*/*v*), although it is clear that conductivity affects hydrolysis, the mechanisms by which this occurs have not been fully understood [22]. On the other hand, ions are known to have both the ability to compete with the enzyme for nearby water molecules and to interact with the enzyme from their position within a layer of water [24]. Therefore, since the reaction was carried out with whole bovine plasma diluted with water to the final protein concentrations tested and considering that the bovine plasma contains approximately 0.1% of inorganic compounds, including calcium, sodium, and magnesium salts, in a second test, the plasma was dialyzed and subsequently hydrolysed under the same conditions (Figure 1B). The results suggest that the compounds removed by dialysis had an important effect on both the rate of hydrolysis, judging by the shape of the curves obtained, and on the DH_max_ values reached.

According to the results obtained and considering that this study aims to evaluate the effect of the substrate concentration on the production of bioactive peptides, which usually show more bioactivity when they are smaller, the following tests were performed on dialyzed bovine plasma due to the higher DH_max_ values obtained.

### 3.2. Effect of Substrate Loading at Fixed E:S Ratio on Peptide Size Distribution and Peptide Profile

To test whether varying the substrate loading at fixed E:S ratio also changed the peptide size distribution, the dialyzed bovine plasma protein concentration was adjusted to the lowest and highest protein concentrations tested in Section 2.1, 5, and 20 g/L, and then hydrolysed with Alcalase using an E:S ratio of 1:25 (*w*/*w*, Alcalase mass per plasma protein mass). Afterwards, the hydrolysis reaction was stopped at several DH values, and the resulting peptide size distributions were analysed by SE-HPLC. These results suggest a faster progression of proteolysis at the lower substrate concentration.

Thus, the relevance of the present design lies in evaluating whether different absolute concentrations of protein and enzyme, applied under the same proportional dosing rule, lead to different hydrolysis outcomes. These findings indicate that substrate loading influenced the progression of proteolysis under a fixed E:S ratio. At the lower substrate concentration, the larger fraction decreased more rapidly at early stages of hydrolysis, whereas the system with higher substrate loading showed a slower reduction in this fraction and a lower accumulation of small peptides at the end of the process. It is clear that, although there is a higher breakage rate of large peptides in the less loaded system, in both cases, when the experiment finished, and the DH value was 19%, a significant proportion of peptides with a size larger than 14 kDa persisted. According to the Linderstrom-Lang theory, for a given DH, the portion of intact protein should be lower for a reaction following the one-by-one mechanism than for one following the zipper mechanism [18]. Considering that a high proportion of large fractions is consistent with the presence of a large amount of intact protein, the behavior of the bovine plasma–Alcalase system suggests the coexistence of multiple kinetic effects. This interpretation is consistent with Adler-Nissen’s general description of proteolytic hydrolysis, in which more than one hydrolysis mechanism may coexist [25], and with later modeling results reported for bovine plasma hydrolysis with Alcalase in this type of system. However, the greater occurrence of smaller peptides in the system with a low substrate loading compared to those with a high loading, indicates a tendency to favour the zipper mechanism under the reaction conditions assessed. Moreover, at DH 19%, there was a significant difference (*p* < 0.05) in the relative area percentage corresponding to the smallest apparent molecular weight fraction (<3 kDa) between both hydrolysates. In the 5 g/L assay, peptides below 3.0 kDa represented 45% of the total chromatographic area, whereas in the 20 g/L assay this fraction represented 35%. The results indicate that substrate concentration influenced the hydrolysis pattern of the bovine plasma–Alcalase system, leading to different peptide distributions despite the same proportional enzyme dosing. The lower proportion of peptides below 3 kDa at 20 g/L compared with 5 g/L suggests that the reaction proceeded through a different cleavage pattern, consistent with a change in the preferential hydrolysis of specific peptide bonds. In this way, the data support the view that substrate concentration affected not only the extent of hydrolysis but also its apparent selectivity [18].

On the other hand, previous studies show that the effect of enzyme dosage on peptide generation is system-dependent. In casein hydrolysis, Tu et al. found that increasing the E:S ratio and hydrolysis time reduced the number of identified peptides [13], whereas Le Maux et al. reported that β-lactoglobulin hydrolysates produced at similar DH values but with different E:S ratios showed comparable molecular mass distributions and DPP-IV inhibitory activity [26]. These studies indicate that the impact of E:S variation depends on the protein system and the hydrolysis conditions. The present work addressed a different issue: whether changing substrate loading at a fixed E:S ratio could still alter peptide size distribution, the peptide profile, and bioactivity. These results show that, at a fixed E:S ratio, substrate concentration can influence both hydrolysis extent and cleavage selectivity in the bovine plasma–Alcalase system, as previously reported for other protein systems [27].

To further examine whether varying the initial substrate concentration influences the peptide profile at a fixed degree of hydrolysis, the peptides identified in the <3 kDa permeates obtained from hydrolyses performed at different protein levels were grouped according to their number of amino acid residues (Figure 2). Together with the quantitative differences observed by SE-HPLC, these results support the idea that understanding the phenomena associated with enzymatic protein hydrolysis requires considering operational parameters such as substrate loading in the reactor. These parameters may influence the apparent selectivity of the enzyme and, therefore, its tendency to hydrolyse certain peptide bonds more rapidly than others, which implies that not all peptide bonds in a protein chain are equally susceptible to attack by the enzyme. This influences the probability associated with the appearance of peptide fragments of a particular length, which may represent the main limitation on producing peptides with specific characteristics.

### 3.3. Effect of Substrate Loading at Fixed E:S Ratio on the Antioxidant Activity of Bovine Plasma Hydrolysates

The difference observed in antioxidant activity among fractions (Table 1) suggests that the initial substrate concentration influenced not only peptide size distribution, but also characteristics of the peptide mixture generated during hydrolysis. The effect of initial substrate concentration on the estimated relative distribution of amino acid residues within identified peptide sequences was examined through the profiles shown in Figure 3. In this regard, the higher relative occurrence of Pro, Gly and Ser in the F20 may be relevant to the higher antioxidant activity measured for this fraction, since these residues have been associated with antioxidant peptides in different protein hydrolysate systems. Among these three amino acids, Pro and Gly are the most abundant in type I collagen, from which many peptides with important antioxidant properties have been identified [28]. Regarding Gly, it increases the flexibility of the peptide backbone and facilitates the exposure of other amino acids with antioxidant properties to free radicals [29]. In this respect, many peptides with antioxidant properties have been identified in the scientific literature that contain a high proportion of this amino acid, such as Gly-Phe-Pro-Ser-Gly [30] or Asn-Gly-Leu-Glu-Gly-Leu-Lys [31], among many others. In particular, Rajapakse et al. [31] found that the presence of Gly played an important role in preventing lipid oxidation. The amino acid proline has also been related to peptides with high antioxidant properties, such as the proline-rich polypeptides isolated from human and bovine colostrum [32] or the peptides obtained after hydrolysing goat milk proteins with pepsin [33]. The presence of Pro in these peptides could produce abrupt conformational changes in their secondary structure, increasing the exposure of other amino acids with antioxidant properties to the medium. Finally, the side chain of Ser contains a primary hydroxyl group, which is a proton-transferring group, able to directly interact with the ABTS or DPPH molecules when it is incorporated within a peptide sequence [34]. Thus, Ser has been found in many peptides with antioxidant properties reported in the scientific literature, such as those from the gastrointestinal digestion of honey protein [35], wheat gluten [36], or peanut protein [37]. Therefore, the higher presence of these three amino acids in the peptides found in F20 may be responsible for the higher antioxidant properties measured for this sample.

To obtain an integrated view of antioxidant performance, the Relative Antioxidant Capacity Index (RACI) [38] was calculated from the ABTS, FRAP, and ORAC results. The RACI values ranked the fractions as F15 > F20 > F10 > F5, indicating that the fractions obtained at higher substrate loadings showed the most favorable overall antioxidant profile. This integrated analysis supports the view that antioxidant performance depended on the assay considered, but also confirms that the hydrolysates produced at 15–20 g/L had the highest combined antioxidant potential. The individual antioxidant assays did not rank the fractions identically. While F20 showed the highest ABTS and ORAC values, F15 presented the highest FRAP value. Therefore, RACI provides a more integrated interpretation of antioxidant performance and supports the conclusion that the hydrolysates obtained at higher substrate loadings (15–20 g/L) had the most favorable overall antioxidant profile.

### 3.4. Role of Substrate Loading at Fixed E:S Ratio in the ACE Inhibitory Potential of Bovine Plasma-Derived Peptides

The in vitro ACE inhibition results and the AHTpin screening shown in Table 2 followed the same general trend among fractions. Therefore, according to the in vitro and in silico results, the peptides obtained when the reactor was loaded with the lowest levels of initial substrate concentration, 5 and 10 g/L, showed higher antihypertensive properties than those obtained when the initial substrate concentration was higher. This may well be because F10 and F5 showed a greater representation of the shortest identified peptides (Figure 2), a characteristic that has been strongly related to a high capacity to inhibit ACE [39]. This relationship between the antihypertensive capacity of peptides and their size has been confirmed by other authors. In this sense, Hyun and Shin [1] hydrolysed BSA with Alcalase and the resulting hydrolysate was ultrafiltered using Amicon membranes with an MWCO of 10, 3, and 1 kDa. In this case, the permeate obtained after the filtration of the hydrolysate with the 1 kDa MWCO membrane showed an IC50 of 0.12 mg/mL, 1.5 times lower than the permeate obtained with the 3 kDa MWCO membrane and almost 4 times lower than the permeate obtained with the 10 kDa MWCO membrane. In addition, Lafarga et al. [3], hydrolysing BSA with papain, also observed an increase in the ACE Inhibition properties of the hydrolysate after serial filtrations with membranes from 10 to 1 kDa MWCO.

In agreement with this trend, the fractions produced at lower substrate concentrations also included several of the peptide sequences with the highest predicted antihypertensive potential. On the other hand, the F20 fraction, which showed a significantly lower in vitro ACE inhibition capacity (*p* < 0.05) compared to the F5 fraction, contained fewer peptides with high inhibitory potential, and most of them had SVM scores below 2.0. According to these results, it seems that there is a positive relationship between the SVM score reported by AHTpin and the in vitro inhibition results.

This behaviour is also consistent with the interpretation proposed above for peptide generation at a fixed E:S ratio. If increasing substrate loading changes the apparent selectivity of cleavage and the relative representation of short peptides [18], then the resulting fractions may differ in ACE-inhibitory capacity even when compared at the same final DH. In the present study, the lower substrate loadings generated fractions with a greater representation of short identified peptides and stronger ACE inhibition, whereas the higher substrate loading produced a distinct peptide population with lower ACE-inhibitory performance.

Among the four peptides analysed, all of which showed an SVM score higher than 2.00 according to the AHTpin platform, as described in Section 2.3, the peptide YNPDIIKVK, found in F15 and F10 but in a relatively low proportion [20], showed the most favourable binding index of −27.5 kcal/mol, while the three remaining peptides were only found in F5 and all showed a similar affinity index: GEVLPLPEANFPSF (−22.6 kcal/mol), VSLPEVPGEY (−21.7 kcal/mol) and VSFTLPRSPTSQE (−21.8 kcal/mol). A search in the BIOPEP-UWM database (https://biochemia.uwm.edu.pl/biopep-uwm/, accessed on 16 March 2026) showed that the identified peptides had not been previously reported, but some tripeptides and dipeptides, such as LPL, PLP, and EY [40], have been reported with ACE activity and can be found within the peptide sequence of GEVLPLPEANFPSF and VSLPEVPGEY.

Docking results were analyzed to explore the binding behavior of the selected peptides toward the ACE active center (Figure 4). The analysis suggests that the interactions between the peptides and the ACE were mainly due to hydrogen bonds and, to a lesser extent, to van der Waals interactions. It has to be considered that in its molecular structure, ACE has three binding pockets: S1, with three residues (Ala354, Glu384, and Tyr523); S2′, which comprises four residues (Gln281, His353, Lys511, and His513); and the S1′ site, which contains the residue Glu162 [41]. In this sense, the peptide YNPDIIKVK efficiently coupled with ACE through 22 hydrogen bonds, some of them formed between carbonyl groups of the amino acids within the peptide sequence and some residues found in the S1, S2′, and S1′ pockets. In addition, according to the proposed model, the amino acid Ile5 directly interacts with the zinc ion found in the ACE active site.

Regarding the sequence VSLPEVPGEY, this peptide interacted efficiently with the active centre of the ACE through 21 hydrogen bonds and two van der Waals bonds. These interactions were especially produced with the residues His353, Tyr520, and His513 in the S2′ pocket; and Ala354 and Tyr523 at the S1 pocket. On the other hand, a serine and a phenylalanine carbonyl group present in the GEVLPLPEANFPSF and VSFTLPRSPTSQE sequences, respectively, interacted with the zinc ion in the ACE active centre through hydrogen bonds. There were also hydrogen bond interactions between these two peptides and the residues Ala354 and Tyr523 at the S1 site, the Tyr523 at the S1 pocket, and His353 at the S2′ pocket.

According to these results, the four peptides studied, three of which are found in F5, have a significant potential to inhibit ACE, judging by the large number and type of interactions produced between these peptides and the active site of the enzyme. These interactions, especially those produced by hydrogen bonds, have an important role in the stabilization of the non-catalytic ACE-peptide complex [42]. In addition, it should be pointed out that all these peptides have proline in their primary structure, a hydrophobic amino acid whose presence and proximity to the C position in the peptide chain have been successfully linked to ACE inhibition [42].

## 4. Materials and Methods

### 4.1. Protein Sample Preparation

Bovine blood was collected immediately after slaughtering from a local slaughterhouse (Asturias, Spain) and poured into 3 L plastic containers. Sodium citrate, previously added at 2% (*w*/*v*), was used as an anticoagulant.

Plasma was separated from the cell fraction by centrifugation for 10 min at 10,000 *g* and 10 °C. The plasma, which is the supernatant resulting from centrifugation, was decanted and stored at −20 °C.

The protein concentration in the plasma fraction was determined by the modified Lowry method [38] using BSA as a standard. A portion of the plasma obtained was dialyzed using a cellulose dialysis membrane with a MWCO of 14,000 Da (D9402-100FT, Sigma-Aldrich, St. Louis, MO, USA). During the dialysis, the distilled water (hereafter, water) was changed periodically to obtain an electrolytic conductivity value below 1 µS (Hanna Instruments HI98129, Woonsocket, RI, USA). The plasma protein concentration was assessed again after the dialysis process.

### 4.2. Enzymatic Hydrolysis

The hydrolysis of the different protein samples was carried out in 100 mL reactors connected to an automatic pH adjustment system (pH-Buret 24 2s, Crison, Alella, Spain) and an isothermal stirrer. Temperature and pH were kept constant at optimal values of 60 +/− 1 °C and 9.0, respectively. Alcalase^®^ 2.9 L (2.4 AU-A/g) of food grade (EC: 3.4.21.14, CALBIOCHEM, Oakville, ON, Canada) was used to perform the enzymatic hydrolysis. Both the raw and the dialyzed plasma were diluted with water to obtain protein concentrations of 5, 10, 15, and 20 g/L and then hydrolysed using an enzyme–substrate ratio of 1:25 (*w*/*w*, Alcalase mass per plasma protein mass) for 2 h. The reaction was stopped by inactivating the enzyme in a heating bath at 90 °C for 5 min. The enzyme dose was scaled proportionally with the protein concentration in order to maintain a constant E:S ratio across treatments. Therefore, both absolute substrate and enzyme concentrations varied simultaneously, while the relative enzyme dose per unit of protein remained constant. This design was selected to compare hydrolysis systems differing in substrate loading without simultaneously introducing variation in the E:S ratio as an additional experimental factor.

In order to follow the reaction process, the pH-stat method was carried out, which is the most commonly used method due to its simplicity. During the reaction, 1.0 N NaOH was added to keep the pH value constant at 9.0. The base added to keep the pH constant neutralizes only the protons released during the breaking of the amide bond of proteins at alkaline pH, which are replaced by the cation of the base. Thus, the protons generated by the hydrolysis are equivalent to the moles of base added [25]. The degree of hydrolysis (DH) was calculated using Equation (1).

(1) $$\mathrm{DH}\;=\frac{h}{h_{\mathrm{tot}}}\;\times \;100=\;\frac{V_{\mathrm{NaOH}}\;\times \;N_{b}}{\mathrm{Mp}\;\times \;\alpha \;\times \;h_{\mathrm{tot}}}\;\times \;100$$

where h is the number of peptide bonds hydrolysed; h_tot_ is the total number of peptide bonds in the substrate (eq/kg); V_NaOH_ is the total NaOH volume (L), N_b_ is the base normality, Mp is the protein mass (in kg) and α is the degree of dissociation of the α-NH2 groups released in the reaction and calculated directly from Equation (2). This parameter α depends on the operating pH and pK. The latter varies significantly with the reaction temperature and can be estimated according to Equation (3). A calculated α value of 0.992 was used according to the pH-stat methodology described by Adler-Nissen [25]. The h_tot_ value used for bovine plasma in this study was 8.3 meq/g, estimated from the amino acid composition of the same substrate determined in our laboratory.

(2) $$\alpha =\frac{10^{pH-pK}}{1+10^{pH-pK}}$$

(3) $$\mathrm{pK}=7.8+\frac{298-T}{298\;\times \;T}\;\times \;2400$$

where T is the temperature in Kelvin.

### 4.3. Peptides Molecular Weight Distribution and Identification

A quantitative analysis of the molecular weight distribution of the peptides found in the hydrolysed plasma was carried out by size exclusion liquid chromatography (SE-HPLC). For that purpose, a high-resolution liquid chromatograph (Agilent 1200, Agilent Technologies Inc., Santa Clara, CA, USA) equipped with a diode array UV detector operating at 214 nm and a size exclusion chromatography (SEC) column (300 × 7.8 mm, Yarra SEC-2000, Phenomenex, Torrance, CA, USA) was used. All samples were filtered through 0.45 µm PVDF filters (Millipore, Burlington, MA, USA) before injection (20 mL), and water and acetonitrile were used as mobile phase in a 90/10 (*v*/*v*) proportion with 0.03% TFA (*v*/*v*). All the samples were eluted with a constant flow of 1 mL/min.

The column was previously calibrated using proteins of different sizes as standards: Ribonuclease A (13.7 kDa), Albumin (44.3 kDa), Thyroglobulin (670 kDa), tri-glycine (0.225 kDa), and Glycine (0.075 kDa). The resulting calibration curve showed a coefficient of determination (R2) of 0.96. In order to analyse the molecular weight distribution of the peptides, Agilent Chemstation software (version B.03.02) was used. The relative area percentage corresponding to each apparent molecular weight interval was used as the quantitative descriptor of peptide size distribution. Statistical comparisons among hydrolysates were performed using these relative area percentages for each apparent molecular weight interval by one-way (ANOVA), followed by Fisher’s least significant difference (LSD) test (*p* < 0.05).

Before peptide identification, hydrolysates were filtered using centrifuge tubes with a MWCO of 3.0 kDa (Macrosep^®^ Advance, Pall Corporation, Port Washington, NY, USA) and 200 μL of the obtained permeates were desalted with a ZipTip C18 column (Millipore, Burlington, MA, USA), 5 μL was recovered, brought to a final volume of 25 μL and analysed by high pressure liquid chromatography coupled to tandem mass spectrometry (RP-UPLC-MS/MS) with a Dionex Ultimate 3000 RS UHPLC (Thermo Fisher Scientific, Waltham, MA, USA) connected on-line to a Bruker Impact II Q-ToF mass spectrometer (Bruker, Billerica, MA, USA). In this case, a Bruker Intensity Trio C18 column (50 × 2.1 mm, 3 µm) was used. Solvent “A” was composed of MilliQ ultrapure water and 0.1% (*v*/*v*) formic acid. Solvent “B” consisted of acetonitrile and 0.1% (*v*/*v*) formic acid, used in a 2% gradient over 1 min, followed by an increase from 2% to 35% over a 30 min period, 35% for 1 min, 35% to 80% for 4 min, and finally 80% B for 1 min.

The injection volume was 2 μL with a flow rate of 150 μL/min. Separation was performed at 30 °C, and mass spectra were obtained in positive ion mode, *m*/*z*. Nitrogen was used as the drying gas at a flow rate of 6.0 L/min and at 300 °C and as the nebulizing gas at a pressure of 0.31 MPa. The capillary voltage was adjusted to 4500 V, and only peaks with intensity greater than 10% of the most intense peak were considered for analysis. PEAKS™ Studio software (v.10.6 Bioinformatics Solutions Inc., Waterloo, ON, Canada; available from Bioinformatics Solutions) was used to identify the peptide masses obtained after LC-MS/MS analysis [43]. In this case, the mass tolerance limit was 0.3 Da, and the minimum peptide extension considered was three amino acids.

### 4.4. Bioactive Potential of Plasma Hydrolysates

As was mentioned in Section 2.2, the plasma protein at 5, 10, 15, and 20 g/L was hydrolysed using Alcalase at an enzyme–substrate ratio of 1:25 (*w*/*w*, Alcalase mass per plasma protein mass) until the DH value reached 19%, and then the reaction was stopped at 90 °C for 10 min. Afterwards, the hydrolysates were ultrafiltered using centrifuge tubes with a MWCO of 3.0 kDa, and the permeates, namely F5, F10, F15, and F20, were obtained depending on the initial plasma protein concentration hydrolysed in each case. Finally, the permeates were lyophilized at 0.1 mBar, −70 °C for 24 h with a Telstar Cryodos device (Telstar, Terrassa, Spain). The protein content in every lyophilized sample was determined by Lowry’s method [44].

#### 4.4.1. Determination of Antioxidant Activity by ABTS

The ABTS antioxidant activity of F5, F10, F15, and F20 was performed following the method described by Re et al. [45]. According to this method, a 100 μL sample of 0.4 mg/mL of protein was mixed with 1000 μL of ABTS solution and then incubated at room temperature for 30 min. The absorbance was recorded at 730 nm in a UV-Vis Genesys™ 10S UV-Vis spectrophotometer (Thermo Scientific, Waltham, MA, USA) using Trolox as a standard. All results were expressed as micromoles of Trolox equivalents per gram of protein (μmol TE/g).

#### 4.4.2. Determination of Antioxidant Activity FRAP

The antioxidant potential of F5, F10, F15, and F20 was also determined by measuring their capacity to reduce ferric iron (Fe^3+^) to ferrous iron (Fe^2+^) according to the procedure described by Pulido et al. [46]. Briefly, 900 µL of FRAP reagent containing TPTZ, FeCl_3_, and 3 M acetate buffer was mixed with 90 µL of distilled water and a 30 µL sample of 0.4 mg/mL of protein and incubated at 37 °C in a dry bath for 30 min. The increase in absorbance at 595 nm was measured using a Genesys™ 10S UV-Vis spectrophotometer (Thermo Scientific, USA) and a sodium acetate buffer as blank. A Trolox solution was also used as a standard. All results were expressed as micromoles of Trolox equivalents per gram of protein (μmol TE/g).

#### 4.4.3. Oxygen Radical Absorbance Capacity ORAC

The oxygen radical absorption capacity of F5, F10, F15, and F20 was measured following the methodology described by Ou et al. [47]. Briefly, 150 µL of 1 µM fluorescein and 25 µL of the sample diluted in 10 µM phosphate buffer at pH 7.4 to a protein concentration of 0.2 mg/mL were mixed. This mixture was preincubated for 30 min at 37 °C, followed by the addition of 25 µL of a 2,2′-azobis (2-methylpropionamidine) dihydrochloride (AAPH) 250 mM solution. Fluorescence intensity was measured at excitation and emission wavelengths of 485 and 520 nm, respectively.

#### 4.4.4. Integrated Antioxidant Index (RACI)

To integrate antioxidant performance across assays, the Relative Antioxidant Capacity Index (RACI) was calculated from the ABTS, FRAP, and ORAC results using the method of Sun and Tanumihardjo. The values from each assay were standardized to z-scores, and the RACI for each fraction was obtained as the mean of these standardized scores. Positive RACI values indicate above-average antioxidant capacity, whereas negative values indicate below-average capacity [38].

#### 4.4.5. Determination of ACE Inhibitory Activity

The ACE inhibitory capacity of the hydrolysates was determined using the spectrophotometric method proposed by Cushman & Cheung [48] with some modifications. One hundred 100 μL of hippuryl–histidyl–leucine solution (5 mM) in tetra-hydrated sodium metaborate (100 mM) and NaCl (300 mM) at pH 8.3 was added to a 40 μL sample of 0.4 mg/mL of protein and 20 μL of ACE at a concentration of 0.1 U/mL. Milli-Q water was used as a blank. The reaction mixture was incubated for 30 min at 37 °C and subsequently stopped by adding 250 μL of 1 M HCl. Afterwards, 1 mL of ethyl acetate was added, and the mixture was centrifuged for 10 min at 1500 *g* and 25 °C to extract the released hippuric acid (HA). The ethyl acetate was subsequently taken out by a micropipette and transferred into a test tube and evaporated at 75 °C for 20 min. The HA was dissolved in Milli Q water, and its concentration was measured at 228 nm using a Genesys™ 10S UV-Vis spectrophotometer (Thermo Scientific, USA). The percentage of inhibition of ACE was calculated as the difference between the absorbance of HA formed after the action of ACE without an inhibitor and the absorbance in the presence of the inhibitor. The IC50 was determined by linear regression testing peptide concentrations from 0.2 to 1.0 mg/mL.

### 4.5. Molecular Docking Calculations

Selected peptide sequences were placed within the ACE active site by means of molecular docking calculations employing the AutoDock CrankPep (version 1.0, ADCP) program, an AutoDock docking engine capable of folding and docking peptide segments with up to 20 amino acids in length. ADCP uses a grid-based representation of the receptor that combines van der Waals, electrostatic, and dehydration contributions, and incorporates intramolecular energies and empirical entropy corrections [49]. In local docking calculations, ADCP achieved the best predictions in a recent benchmark analysis of 14 docking programs [50].

Starting coordinates for the ACE enzyme were obtained from the 1O86 crystal structure. All the water molecules and the lisinopril inhibitor were removed, and residues Leu433 and Asp440 were capped with N-methyl and acetyl terminal residues, respectively, to replace the unresolved sequence. Other missing atoms and hydrogens were added with the tLeap program and structurally relaxed using the SANDER program included in the Amber21 suite. To improve the accuracy of the scoring function for zinc enzymes, a fractional charge (+1.19) was assigned to the zinc atom in the ACE active site. This value corresponds to the B3LYP/6-31G(d) electrostatically fitted atomic charge of the Zn ion in a small cluster model, [Zn(methyl-imidazole)2(acetate)]+, extracted from the 1O86 crystal structure and augmented with H-atom coordinates. Only the H atoms were relaxed at the B3LYP/6-31G(d) level of theory [51] and using the Gaussian09 program [52]. To preserve charge integrity, a +0.81 charge was distributed evenly throughout the Zn-coordinated residues. Other docking parameters presented default values. For each peptide, an initial coil conformation was selected to run 64 independent ADCP Monte Carlo simulations (replicas) with 2.5 million evaluations of the scoring function per amino acid and replica. The grid affinity maps were computed in a docking box centred at the position of the zinc ion that extended 30 Å along each dimension.

### 4.6. Statistical Analysis

Experiments were performed in triplicate and are shown as the mean value ± standard deviation. Data were analysed by one-way analysis of variance (ANOVA), and mean comparisons were performed using Fisher’s least significant difference (LSD) test at a significance level of *p* < 0.05. These analyses were performed using Statgraphics^®^ V.15.2.06 statistical software.

## 5. Conclusions

Increasing the initial protein concentration in the reactor, while keeping the enzyme–substrate ratio constant, led to a mixture of peptides with a size distribution shifted towards larger peptides and with a slightly different estimated relative distribution of amino acid residues, probably due to changes in the enzyme selectivity. Consequently, the hydrolysates showed different antioxidant and antihypertensive capacities. In this sense, the hydrolysate with the smallest peptides showed higher antihypertensive properties, since these peptides were able to interact with the narrow active centre of the ACE, but on the other hand, the hydrolysate with the highest concentration of Pro, Gly, and Ser possessed higher antioxidant properties.

According to the results obtained, if the objective is to obtain peptides with the smallest size possible, it is more appropriate to perform the Alcalase hydrolysis of the plasma proteins after the dialysis of the bovine plasma. The nature of the inhibitors present in the raw plasma has not been resolved, although, owing to the relatively high presence of salts in the plasma and given their capacity to influence the rate of enzymatic reactions, they may be responsible for such a decrease in the hydrolysis performance.

Overall, changes in the initial concentration of the substrate have been proven to be an easy and affordable way to modulate the bioactivity of the hydrolysates obtained.

## Figures and Tables

**Table 1:** In vitro antioxidant activity (ABTS, FRAP, and ORAC) of the peptides found in the permeates (MWCO 3.0 kDa) obtained from the bovine plasma–Alcalase hydrolysates (DH = 19%) with S_0_ = 5, 10, 15 and 20 g/L (F5, F10, F15, and F20, respectively). It was determined using the methods described in Section 4.4.

Fraction	Antioxidant Activity μmolTE/g Protein						
	ABTS		FRAP		ORAC		RACI *
F20	1833.98	±78.54 ^a^	304.03	± 25.99 ^b^	1461.36	±70.17 ^a^	0.496
F15	1719.01	±171.30 ^a^	347.25	±21.28 ^a^	1302.99	±78.77 ^b^	0.618
F10	1596.85	± 138.25 ^b^	303.74	± 18.20 ^b^	1389.08	± 56.97 ^b^	−0.186
F5	1493.13	±101.41 ^b^	310.95	±28.45 ^a,b^	1071.63	±58.15 ^c^	−0.929

* Superscript letters indicate significant differences between fractions within assays (*p* < 0.05). RACI: Relative Antioxidant Capacity Index, calculated as a composite antioxidant index from the standardized ABTS, FRAP, and ORAC values.

**Table 2:** Antihypertensive potential of fractions F5, F10, F15, and F20 obtained from bovine plasma–Alcalase hydrolysates prepared at initial protein concentrations (S_0_) of 5, 10, 15, and 20 g/L, respectively. The table includes the number of individual peptides with AHTpin scores ≥ 0.9 and <0.9 in each fraction, together with ACE inhibition (%) and IC50 values, determined as described in the Materials and Methods Section.

FRACTION	Num. Peptides AHTpin ≥ 0.9	Num. Peptides AHTpin < 0.9	% ACE Inhibition	IC_50_ (mg/mL)
F5	73	331	53.73 ± 3.08 ^a^	0.41
F10	132	369	47.00 ± 1.27 ^b^	0.66
F15	62	311	38.30 ± 1.21 ^c^	0.65
F20	23	56	35.96 ± 1.55 ^c^	0.86

Superscript letters indicate significant differences between fractions within assays (*p* < 0.05).

## Data Availability

The original contributions presented in this study are included in the article. Further inquiries can be directed to the corresponding author.

## Abbreviations

ABTS	2,2′-azinobis(3-ethylbenzothiazoline-6-sulfonic acid)
ACE	angiotensin-converting enzyme
ADCP	AutoDock CrankPep
AHTpin	plataforma para predicción de péptidos antihipertensivos
ANOVA	analysis of variance
AAPH	2,2′-azobis(2-methylpropionamidine) dihydrochloride
BIOPEP-UWM	base de datos BIOPEP-UWM de péptidos bioactivos
BSA	bovine serum albumin
DH	degree of hydrolysis
FRAP	ferric reducing antioxidant power
IC50	half-maximal inhibitory concentration
LSD	least significant difference
MWCO	molecular weight cut-off
ORAC	oxygen radical absorbance capacity
PDB	Protein Data Bank
PEAKS	PEAKS Studio software
PVDF	polyvinylidene fluoride
QSAR	quantitative structure–activity relationship
RACI	relative Antioxidant Capacity Index
